# Full-Length Genomic Sequence of Subgenotype IIIA Hepatitis A Virus Isolate in Republic of Korea

**DOI:** 10.1155/2013/426034

**Published:** 2013-08-07

**Authors:** Ah-Ra Lee, Sung-Geun Lee, Lae-Hyung Kang, Weon-Hwa Jheong, Soon-Young Paik

**Affiliations:** ^1^Department of Microbiology, College of Medicine, The Catholic University of Korea, Seoul 137-701, Republic of Korea; ^2^Korea Zoonosis Research Institute, Chonbuk National University, Jeonju 561-756, Republic of Korea; ^3^Environmental Infrastructure Research Department, National Institute of Environmental Research, Incheon 404-708, Republic of Korea

## Abstract

Hepatitis A virus is known to cause acute hepatitis and has significant implications for public health throughout the world. In the Republic of Korea, the number of patients with hepatitis A virus infection has been increasing rapidly since 2006. In this study, the Kor-HAV-F strain was identified as subgenotype IIIA by RT-PCR, and its identity was confirmed by nucleotide sequencing and alignment analysis. Moreover, detailed phylogenetic analysis indicated that the Kor-HAV-F strain clustered into subgenotype IIIA, including strains isolated in Japan, Norway, and India. The entire amino acid sequence of the VP1 and 2A regions was compared with that of the reference strains isolated in various countries. We found 2 amino acid changes (T168A and L96P, resp.) in the VP1 and 2A regions, which had not been found in any other hepatitis A virus strain. To our knowledge, this study is the first to report the full-length sequence of a hepatitis A virus isolated in the Republic of Korea.

## 1. Introduction

Hepatitis A virus (HAV), known to cause acute hepatitis, has significant implications for public health worldwide. HAV infection is endemic in developing countries, including Thailand, India, and Mexico. In contrast, industrialized countries have a decreasing exposure rate to HAV, due to improvements in hygiene and sanitation conditions [1]. Direct person-to-person spread by the fecal/oral route is the most important mean of transmission of hepatitis A, and infection with HAV can cause sporadic and epidemic acute hepatitis in humans [2, 3].

HAV is the only member of the genus *Hepatovirus* within the family Picornaviridae [4]. The positive-sense single-stranded RNA consists of a highly conserved 5′-nontranslated region (NTR), a single open reading frame (ORF) encoding a polyprotein, and a 3′-NTR [4, 5]. The single ORF is divided into 3 functional regions, namely, P1, P2, and P3. The P1 region encodes the capsid polypeptides VP4, VP2, VP3, and VP1, whereas P2 and P3 encode the nonstructural polypeptides (2A–2C and 3A–3D, resp.) [6]. 

Thus far, HAV strains have been classified into 3 human and simian genotypes (I–VI), of which genotypes I, II, and III are found in humans; these genotypes are further divided into subgenotypes IA and IB, IIA and IIB, and IIIA and IIIB, respectively [7, 8]. Most of the human HAV strains belong to genotypes I and III [8–10]. An HAV genotype is defined as a group of viruses with >85% nucleotide sequence identity. The HAV genotypes are further classified into subgenotypes with sequence variability of <7.5% [11].

Subgenotypes IA and IB are most often reported in Brazil, France, China, and Japan [12–14]. Subgenotype IA is the most common type worldwide, whereas subgenotype IB has been prevalent in the regions of Europe, Australia, and Mediterranean [15–18]. Subgenotype IIIA has been found in various countries in Asia, Europe, and USA, while subgenotype IIIB was found to be responsible for some cases of HAV infection in Denmark and Japan [9, 13, 19–23].

Previouse studies on HAV genotypes in Republic of Korea have shown a distinct changing pattern in circulating HAV genotypes over the past 10 years [24]. Until 2004, subgenotype IA was the most prevalent in the Republic of Korea [25]. However, studies conducted between 2004 and 2008 reported the cocirculation of the 2 prevalent subgenotypes IA and IIIA [22, 25, 26]. Subgenotype IIIA has been the predominant subgenotype since 2008, according to a previous study [22, 26].

In this study, the whole genome sequence of a South Korean HAV subgenotype IIIA isolate was analyzed and compared with that of available reference strains to determine the genetic relationship along the entire genome in the Republic of Korea.

## 2. Material and Methods

### 2.1. Stool Sample Collection

An HAV-positive stool sample was isolated from a 35-year-old female patient with fever and myalgia, in Seoul, the Republic of Korea, in October 2011. The sample was obtained from the Waterborne Virus Bank (Seoul, the Republic of Korea). The stool sample was stored at −70°C.

### 2.2. Viral RNA Extraction

The stool sample was diluted to a ratio of 1 : 10 in phosphate buffered saline (PBS), mixed, and centrifuged. From 140 *μ*L of this diluted stool sample, viral RNA was extracted using a QIAamp viral RNA mini kit (Qiagen, Hilden, Germany) according to the manufacturer's instructions. A 50-*μ*L volume of elute was obtained and stored at −70°C until analysis.

### 2.3. Reverse Transcription-PCR (RT-PCR)

For the detection of HAV, reverse transcription polymerase chain reaction (RT-PCR) was performed with a OneStep RT-PCR Kit (Qiagen, Hilden, Germany), with HAV-F and HAV-R primers based on the sequence of the VP1-2A junction region (Table 1). To facilitate sequencing of the entire genome of the detected HAV strain, RT-PCR was performed with the OneStep RT-PCR Kit (Qiagen, Hilden, Germany), with 13 pairs of newly designed primer sets (Table 1). We used 5 *μ*L of viral RNA as template and 20 *μ*L of the premixed kit solution. The PCR was carried out in a PCR System S1000 thermal cycler (BIO-RAD, CA, USA) according to the following protocol: an initial RT step at 50°C for 30 min, followed by PCR activation at 95°C for 15 min and 40 cycles of amplification, each consisting of 1 min at 95°C, 1 min at 52°C to 54°C, and 1 min at 72°C, with a final extension step of 10 min at 72°C. The PCR products were then electrophoresed on a 2% ethidium bromide-stained agarose gel.

### 2.4. Cloning and Sequencing of RT-PCR Products

The amplified fragments were purified from the gel using the HiYield Gel/PCR DNA Extraction Kit (RBC, Taipei, Taiwan). These products were then cloned into the pGEM-T Easy Vector (Promega, Madison, WI, USA) according to the manufacturer's recommendations and transformed into competent *E. coli* DH5*α* cells (RBC, Taipei, Taiwan). Transformants were selected on Luria-Bertani (LB) agar media (Duchefa, Haarlem, The Netherlands) containing 50 *μ*g/mL ampicillin. Clones were expanded overnight at 37°C in 2 mL LB media containing 50 *μ*g/mL ampicillin, centrifuged at 4°C for 10 min at 800 ×g, resuspended in 600 *μ*L fresh LB media with 10% glycerol, and stored at −80°C until required for further use. Plasmid DNA was purified using the HiYield Plasmid Mini Kit (RBC, Taipei, Taiwan) according to the manufacturer's recommendations. DNA was sequenced by Cosmo Genetech (Seoul, the Republic of Korea).

### 2.5. Sequence and Phylogenetic Analysis

The sequence data of the composite sequences of the 13 plasmids were aligned using the Clustal W method with the DNASTAR software (DNAStar, Inc., Madison, WI, USA) and CLC Main Workbench Program version 6.7.1 (CLC Bio, Katrinebjerg, Denmark) to obtain the entire genome sequence. Dendrograms were constructed using the neighbor-joining method with MEGA software version 4.0.

### 2.6. Nucleotide Sequence Accession Number

The nucleotide sequence of the HAV-positive stool sample isolate was submitted to the GenBank database under the following accession no.: JQ655151.

## 3. Results and Discussion

Globally, 1.4 million of patients affected by HAV infection have been reported annually. The prevalence rate of HAV in different countries varies with income and hygiene levels. According to data from Korea Centers for Disease Control and Prevention (KCDC), HAV infection in the Republic of Korea has been increasing consistently since 2001. Moreover, the number of patients with HAV infection has increased more rapidly since 2006. In 2009, the number of patients with HAV infection was approximately 143 times higher than that in 2001. 

Generally, HAV IA was known as major subgenotype in the Republic of Korea. In 1994, 100% of HAV strains were subgenotype IA although this prevalence significantly decreased from year to year, reaching 0% in 2008, whereas HAV IIIA was first detected in 2005 and exhibited a peak prevalence of 100% in the Republic of Korea in 2006 [22, 25–28]. However, the 2 prevalent subgenotypes IA and IIIA cocirculated in the Republic of Korea since 2005 [25]. The difference in circulating subgenotypes differs depending on the region and period. Furthermore, 5 outbreaks associated with HAV infection occurred since 2005. Among them, 3 outbreaks were associated with subgenotype IIIA [29]. Despite their clear importance, complete genome analysis of subgenotype IIIA has not been yet reported in the Republic of Korea.

In this study, we examined the nucleotide and amino acid similarities and phylogenetic tree analysis between the partial and complete sequences of HAV reference strains. The Kor-HAV-F isolate had a genomic length of 7386 nucleotides (nt), excluding the poly(A) tract at the 3′ terminus, and was similar to those of the reported HAV isolates for which the entire genomic sequence is known. Moreover, the isolate possessed a single long ORF of 6684 nt that encoded a polyprotein of 2228 amino acids. The single ORF was divided into 3 functional regions termed P1 (2373 nt), P2 (1893 nt), and P3 (2418 nt).

**Figure 1:**
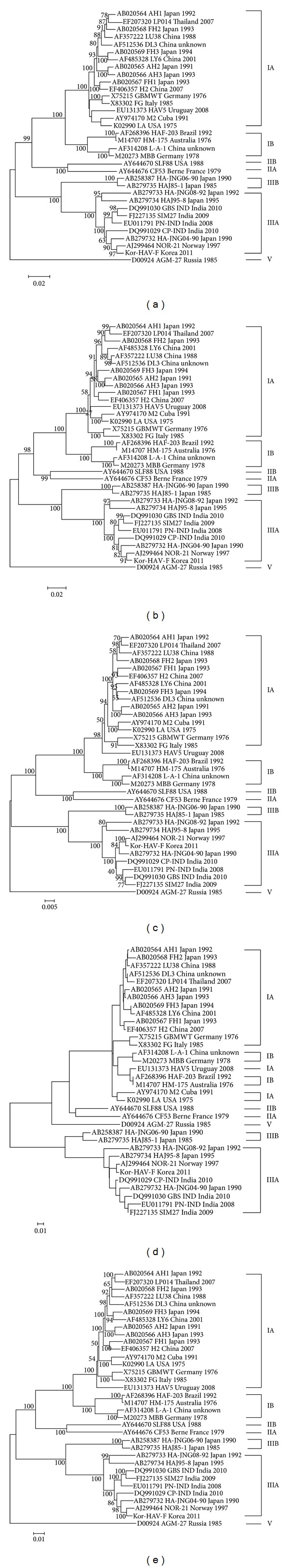
Phylogenetic trees of the nucleotide sequences of HAVs. The phylogenetic tree analysis was based on the nucleotide sequence3 of the entire genomic region (a), P1 region (b), P2 region (c), P3 region (d), and P3A region (e) of the Kor-HAV-F and reference strains.

To assess the genetic relationship between the Kor-HAV-F strain and the other reference strains isolated worldwide, the sequences of the ORFs and the whole genomes were subjected to multiple sequence alignment analysis and phylogenetic analysis (Table 2). Sequence comparison with 33 known HAV isolates revealed that the Kor-HAV-F strain shares the greatest identity with the NOR-21 HAV strain (AJ299464), which was isolated from Norway, with 98.8% identity at nucleotide and 99.9% identity at amino acid sequence levels. The Kor-HAV-F strain clustered with the NOR-21, HA-JNG04-90F, and CP-IND strains in a monophyletic branch. The 3 and 2 strains were isolated from India and Japan, respectively, belonged to 2 distinct clusters within subgenotype IIIA. The identities of the Kor-HAV-F strain with other subgenotypes (1A, IB, IIA, IIB, IIIB, and V) were within the range of 80.6–88.6% (nucleotide identity) and 92–98.5% (amino acid identity), respectively (Figure 1(a)). Similarly, in the phylogenetic analysis of the P1 and P2 regions, the Kor-HAV-F strain clustered with those strains (Figures 1(b) and 1(c)). However, in the case of the P3 region, the Kor-HAV-F strain clustered with the NOR-21 and HA-JNG04-90F strains in a monophyletic branch (Figure 1(d)). Partial phylogenetic analysis of P3A indicated that the Kor-HAV-F strain clustered with only the NOR-21 strain in a monophyletic branch (Figure 1(e)).

**Figure 2:**
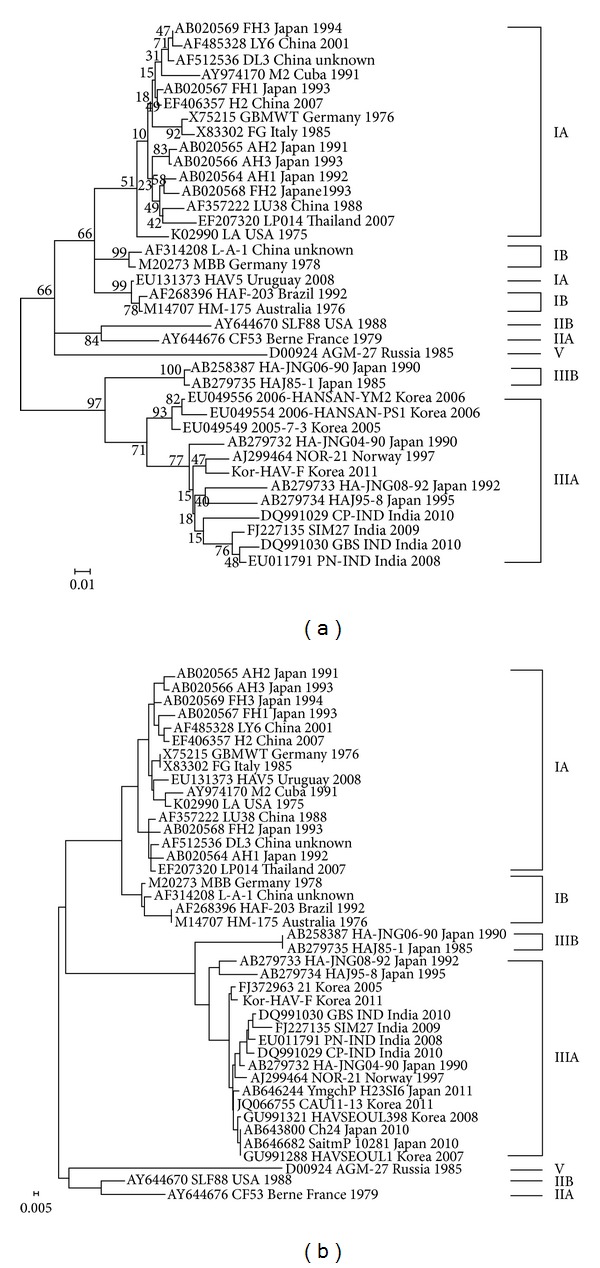
Phylogenetic trees of the nucleotide sequences of HAVs. The phylogenetic tree analysis was based on the nucleotide sequences of the VP3/VP1 (a) and VP1/2A (b) regions of the Kor-HAV-F and reference strains.

Additionally, to assess the genetic relationship between Kor-HAV-F and the Korean strains, the sequences of the VP3/VP1 and VP1/2A junctions were subjected to multiple sequence alignment and phylogenetic analysis. In the case of VP3/VP1, sequence comparisons revealed that the Kor-HAV-F strain shares the greatest identity with the NOR-21 strain (96.7% nucleotide identity), whereas sequence identity with the Korean strains was relatively low (92.9–93.4% nucleotide identity). These Korean strains belonged to a distinct cluster within the subgenotype IIIA (Figure 2(a)). In the case of VP1/2A, sequence comparisons revealed that the Kor-HAV-F strain shares the greatest identity with the 21 (FJ372963) strain isolated in 2005 (98.8% nucleotide identity). The Kor-HAV-F strain clustered with the Korean strains, other than the Korean and Japan strains, in a monophyletic branch (Figure 2(b)). 

Nucleotide and amino acid identities between Kor-HAV-F and representatives of each genotype are shown in Table 1. Kor-HAV-F sequence identity with the other genotypes was in the range of 79%–89.8%, except for subgenotype IIIA in the coding region P1–P3, whereas amino acid sequences differed to a greater extent (89.4–99.1% identity). Compared with subgenotype IA, Kor-HAV-F showed higher identity (82.8–83.3% at nucleotide level; 92.3–97% at amino acid level) with the H2 strain (isolated in 2007, subgenotype IA) than with the GBM strain, isolated in 1976, (81.7–83.1% at nucleotide level; 91.9–96.7% at amino acid level), at coding regions P1 and P3. However, Kor-HAV-F showed higher identity with the older strains than with the recent strains belonging to the subgenotype IB. Among the P1–P3 coding regions, the P3A site had the highest variability at nucleotide and amino acid levels.

**Figure 3:**
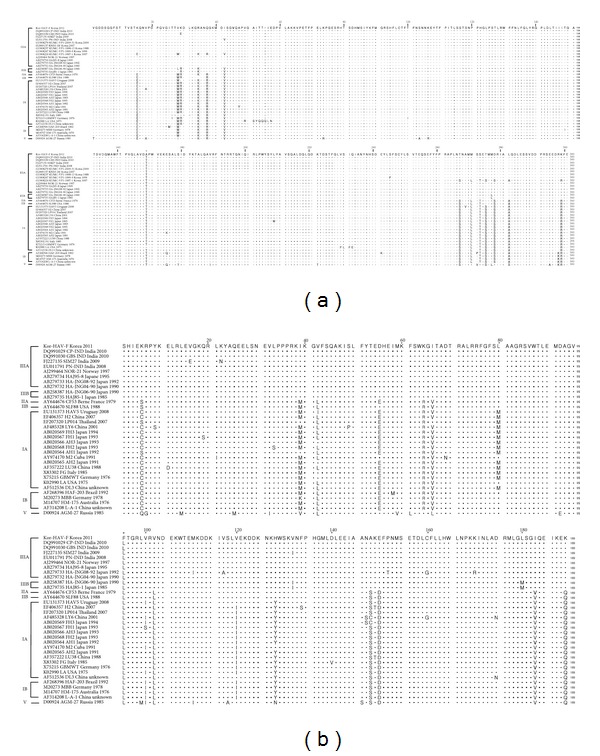
Alignment analysis of the VP1 (a) and 2A (b) regions of HAV strains (asterisk indicated mutation) Amino acid sequences of the VP1/2A region were compared with diverse subgenotype strains reported from various countries, including the Republic of Korea.

Amino acid sequences of the VP1 region (300 amino acids) were compared with diverse subgenotype strains reported from various countries, including the Republic of Korea. The Kor-HAV-F strain showed a distinct substitution of Asn instead of Thr at position 168, which was not found in any other subgenotype. The Kor-HAV-F strain showed 92–99.7% amino acid variation within the P1 region. Only the Kor-HAV-F strain, including subgenotypes IIIA, showed 2 amino acid changes (K34R and V42I) in the P1 region. Furthermore, genotypes III commonly showed 10 amino acid changes (E15K, I/M28L/V, R37Q, S266T, L270 M, T272S, S274T, S277D, A281L, and R298 K; Figure 2(a)). Inconsistent with previous reports, we found 12 amino acid changes in the VP1 region, but consistent with previous reports for other HAV subgenotype IIIA strains, our results also showed the existence of C-terminus cleavage sites as Leu 264/265 Asn, Glu 273/274 Thr, and Glu 285/286 Ser in the HAV VP1 protein (Figure 3(a)).

In the case of the 2A region (189 amino acids), amino acid sequences were compared with diverse subgenotypes reported from various countries. The Kor-HAV-F strain showed a distinct substitution of Phe for Leu at position 96, which was not found in any other subgenotype. The Kor-HAV-F strain showed 87.3–99.5% amino acid variation within the P1 region. Only the Kor-HAV-F strain, including subgenotypes IIIA, showed a single amino acid change (S/C148A) in the 2A region. Furthermore, genotype III commonly showed 8 amino acid changes (K/M/V39I, L42V, E55D, R64 K, L/V66I, D150E, V183I, and Q189 K). In particularly, genotypes II and III showed a single amino acid change at N/Y128H (Figure 3(b)).

In the Republic of Korea, 4 patients with HAV subgenotype IIIA have travelled overseas before the onset of symptoms, according to a previous study. Similarly, the HAV patient in this study had visited Taiwan before the onset of symptoms. Moreover, we found 2 novel amino acid changes that had not been reported in Korea earlier. Hence, it is assumed that this patient probably acquired the virus in Taiwan.

Korea, until the 1980s, was classified as a high endemic country, and most infections occurred in childhood. However, opportunities of infections for exposure decreased, with improvements in sanitation and socioeconomic conditions; therefore, susceptible populations of the infection is changed to adolescents and young adults [18, 30, 31].

Since 2001, the incidence of acute hepatitis was officially reported through the national sentine surveillance system of Korean Center for Disease Control and Prevention; after that, the incidence of acute hepatitis was increased steadily, and it was sharply raised from 2006 [32].

This is the first study reporting the full-length sequence of an HAV isolated in the Republic of Korea. This sequence will be useful for comparison with the full-length HAV sequences of other strains currently identified globally and in future. The whole-genome sequence data derived in this study may prove useful not only for more accurate diagnoses of HAV, but also for basic research relating to the elucidation of genetic functions. Furthermore, it may prove useful for the prediction of newly appearing variants via comparison with HAV strains worldwide, in fundamental research for vaccine development, and eventually, in the field of public health, with identification of new emerging strains of HAV.

## 4. Conclusions

This study, the first to report the full-length sequence of a HAV isolated in the Republic of Korea, is meaningful as it provides a full-length HAV sequence standard for future evolutionary studies. It may also prove useful in the field of public health by facilitating the diagnosis and predicting new emerging variants. Further characterization of full-length sequences of diverse HAV strains circulating worldwide is needed.

## Figures and Tables

**Table 1:** Primer sets used in this study.

	Primer	Sequence (5′ → 3′)	Polarity	Region
Diagnosis primer sets	HAV-F	GGT TTC TAT TCA GAT TGC AAA TTA	+	2873–2889
	HAV- R	AGT AAA AAC TCC AGC ATC CAT TTC	−	3357–3380

Designed primer sets	HAV-1F	GCC TAG GCT ATA GGC TAA AT	+	79–98
	HAV-1R	CGT TCC CAA CAT CTG TGT	−	304–321
	HAV-2F	GTT GTA AAT ATT AAT TCC TGC AGG	+	124–147
	HAV-2R	CAG ACA ATC CAC TTA ATG CAT	−	510–530
	HAV-3F	CTA TGA AGA GAT GCT TTG GAT	+	412–432
	HAV-3R	TGT ATC TCA ATT CCA AAT CTT GC	−	1095–1117
	HAV-4F	ATT CAT TCT GCT GAY TGG TTG	+	972–992
	HAV-4R	CAA CTG GRA TAA CCT TGA TCT	−	1675–1695
	HAV-5F	AGG AAG ATT GGA AAT CTG ATG	+	1549–1569
	HAV-5R	TTC ACT GTT GTA ATR CCA ACT TG	−	2253–2275
	HAV-6F	CAA GTT GGC ATT ACA ACA GTG	+	2253–2273
	HAV-6R	GAG CAA TTC TAT CCA TCA TTG	−	3010–3030
	HAV-7F	GAA ATG GAT GCT GGA GTT TTT ACT	+	3357–3380
	HAV-7R	CTG AAC ARA TAT CYC TAA GCC	−	3991–4011
	HAV-8F	GTT GAG AGT GAT GAA TTA TGC	+	3851–3871
	HAV-8R	YTG TCC ACT ATA TCC ATC CCA	−	4521–4541
	HAV-9F	AAT GGT GMC AAG ATG TGA GCC	+	4364–4384
	HAV-9R	AAC TGC AAC CCA CTT RTG RTT	−	5091–5111
	HAV-10F	GGG ATT ATC AGA TGA TGA CAA	+	4982–5002
	HAV-10R	TAC CTC TCC ARG CTT GAT CAA	−	5743–5763
	HAV-11F	GGA CTC CAA TGT TAA TTT CAG	+	5647–5667
	HAV-11R	TCC ATA TTR ATT GCA TCT ATC CC	−	6234–6256
	HAV-12F	GAT GAG CCA GAT GAT TAT AAA GA	+	6120–6142
	HAV-12R	AGA AGG CAT TGA MCC ACA TAC	−	6819–6839
	HAV-13F	GTA TGT GGK TCA ATG CCT TCT	+	6819–6839
	HAV-13R	WAT TTA CTG AAA AGA YAA AAT AAA CAA AC	−	7436–7466

**Table 2:** Percent identity between the Kor-HAV-F and the strains belonging to other genotypes, at the nucleotide and amino acid levels.

Protein	IA				IB				IIA		IIB		IIIA				IIIB				V	
	GBM/WT		H2		HM-175		HAF-203		CF53/Berne		SLF88		NOR-21		GBS-IND		HAJ85-1		HA-JNG06-90F		AGM-27	
	(1976)		(2007)		(1976)		(1992)		(1979)		(1988)		(1997)		(2010)		(1985)		(1990)		(1985)	
	NT	AA	NT	AA	NT	AA	NT	AA	NT	AA	NT	AA	NT	AA	NT	AA	NT	AA	NT	AA	NT	AA
P1	**83.1**	**96.7**	**83.3**	**97.0**	**83.0**	**97.2**	**82.9**	**96.6**	**82.4**	**97.0**	**82.8**	**97.3**	**98.4**	**99.9**	**95.2**	**99.1**	**89.0**	**99.1**	**88.7**	**99.0**	**80.7**	**94.8**
VP4	95.7	**95.7**	95.7	**95.7**	95.7	**100.0**	95.7	**100.0**	95.7	**100.0**	94.2	**100.0**	**99.6**	**100.0**	98.6	**95.7**	97.1	**100.0**	**97.1**	**100.0**	94.2	**95.7**
VP2	84.8	**98.0**	84.6	**97.6**	84.0	**98.4**	84.0	**98.0**	83.6	**96.7**	83.5	**97.6**	**98.3**	**100.0**	95.4	**99.6**	89.7	**100.0**	**88.0**	**99.1**	82.8	**85.5**
VP3	82.6	**98.2**	83.2	**98.6**	82.7	**98.2**	82.7	**98.2**	81.2	**98.6**	82.6	**98.6**	**98.9**	**100.0**	94.1	**99.1**	88.3	**99.1**	**89.2**	**100.0**	81.4	**97.7**
VP1	81.2	**94.7**	81.3	**95.3**	81.4	**95.3**	81.2	**94.0**	81.3	**95.7**	81.7	**96.0**	**98.1**	**99.7**	95.4	**99.0**	88.4	**98.3**	**88.3**	**98.0**	77.4	**92.0**
P2	**81.4**	**93.2**	**80.8**	**93.2**	**81.2**	**93.3**	**81.0**	**92.4**	**81.9**	**92.6**	**82.3**	**94.0**	**98.5**	**99.9**	**95.6**	**99.5**	**86.8**	**98.4**	**86.7**	**99.0**	**79.0**	**89.4**
P2A	81.7	**92.6**	80.1	**91.0**	80.6	**92.1**	80.6	**91.5**	82.5	**93.7**	82.2	**94.2**	**98.1**	**99.5**	94.7	**98.9**	86.2	**97.9**	**86.4**	**97.9**	78.0	**87.3**
P2B	80.7	**94.4**	81.9	**95.3**	81.0	**95.3**	80.4	**92.5**	81.6	**91.6**	82.6	**93.5**	**98.8**	**100.0**	93.1	**100.0**	88.5	**99.1**	**87.2**	**99.1**	80.1	**91.6**
P2C	81.5	**93.1**	80.8	**93.7**	81.7	**93.4**	81.5	**92.8**	81.7	**92.2**	82.2	**94.0**	**98.7**	**99.7**	96.9	**99.7**	86.7	**98.5**	**86.7**	**98.5**	79.2	**89.9**
P3	**81.7**	**91.9**	**82.8**	**92.3**	**82.7**	**92.0**	**82.7**	**91.8**	**82.6**	**91.8**	**82.4**	**92.0**	**99.3**	**100.0**	**96.9**	**99.9**	**89.7**	**98.0**	**89.8**	**98.0**	**81.9**	**91.4**
P3A	74.8	**79.7**	75.7	**85.1**	75.7	**85.1**	75.7	**95.1**	76.9	**84.7**	76.1	**85.1**	**99.1**	**100.0**	94.1	**100.0**	87.4	**93.2**	**88.3**	**91.9**	75.5	**86.1**
P3B	79.7	**95.7**	79.7	**100.0**	78.3	**100.0**	76.8	**95.7**	87.0	**100.0**	85.5	**100.0**	**100.0**	**100.0**	95.7	**100.0**	85.5	**100.0**	**85.5**	**100.0**	82.6	**95.7**
P3C	83.1	**97.3**	84.3	**97.7**	84.2	**97.7**	84.2	**97.7**	84.3	**98.6**	84.3	**97.7**	**99.1**	**100.0**	96.7	**100.0**	89.5	**100.0**	**90.0**	**100.0**	81.8	**96.4**
P3D	82.2	**90.0**	83.4	**90.8**	83.4	**90.4**	83.4	**90.2**	82.5	**89.6**	82.3	**90.4**	**99.4**	**100.0**	97.5	**99.8**	90.3	**97.8**	**90.2**	**98.0**	82.6	**89.8**
